# Adhesion of Pathogenic Bacteria to Food Contact Surfaces: Influence of pH of Culture

**DOI:** 10.1155/2011/972494

**Published:** 2010-10-11

**Authors:** Akier Assanta Mafu, Corinne Plumety, Louise Deschênes, Jacques Goulet

**Affiliations:** ^1^Food Research and Development Centre, Agri-Food and Agriculture Canada, 3600 Casavant Boulevard-West, St-Hyacinthe, QC, Canada J2S 1A2; ^2^Department of Science and Nutrition, Laval University, Quebec, QC, Canada G1K 7P4

## Abstract

The adhesion of *Aeromonas hydrophila, Escherichia coli* O157:H7, *Salmonella* Enteritidis, and *Staphylococcus aureus* to hydrophobic and hydrophilic surfaces in cultures with different pHs (6, 7, and 8) was studied. The results indicated that the type of material had no effect on the attachment capacity of microorganisms, while environmental pH influenced the adhesion of *A. hydrophila, E. coli,* and *S. aureus* to both solid substrates. The attachment of *S.* Enteritidis (*P* > .05) was not affected by the type of substrate or the culture pH, whereas *E. coli* displayed the weakest affinity for both polystyrene and glass surfaces. No correlation was established between the physicochemical properties of the materials, or the bacterial and the rate of bacterial adhesion, except for *S. aureus*. Photomicrographs have shown that surfaces were contaminated by small clusters of *S.* Enteritidis while *S. aureus* invaded the food contact surfaces in the form of small chains or cell aggregates.

## 1. Introduction

In food processing plants, residues of all kinds chemical, biological, organic, or inorganic inevitably accumulate on the surfaces of equipments in contact with food [1]. Attachment of undesirable microorganisms to these surfaces is a source of concern, since this can result in product contamination leading to serious economic and health problems [2–4]. In fact, this microbial contamination has two components: first, the saprophytic flora responsible for food spoilage and second, the pathogenic flora, which cause infections in humans and animals. To adversely affect the sensory, physical, and chemical qualities of food, a large population of spoilage-causing microorganisms is required, while in the case of food pathogens it only takes a few cells to affect product safety and cause food poisoning.

In the phenomenon of bacterial adhesion to inert surfaces, the physicochemical properties (hydrophobicity and charges) and substrates or surface topography are playing important roles [5–7]. Joints such as valves and any other difficult-to-reach spaces are the most favourable areas to bacterial adhesion. The effect of corrosion on solid materials must also be considered since it can lead to the formation and expansion of cavities and grooves [8]. This in turn provides breeding sites for microorganisms, thereby compromising the efficacy of cleaning and disinfection procedures. The surface characteristics of the microorganisms themselves and the various environmental conditions encountered in agri-food industries (organic materials, pH, temperature, water activity, etc.) also influence microbial attachment to inert surfaces [2, 9–12].

Once they have adhered to inert surfaces, the microorganisms may exhibit a greater degree of resistance to the chemical or natural cleaning and disinfecting agents used in the agri-food industries compared to bacteria in suspension [13, 14]. 

The potential for attachment and development of microorganisms on inert surfaces as well as the resistance of the resulting sessile cells has been and continues to be extensively studied [15–19]. Although an understanding of the parameters that govern the adhesion of these bacteria to solid surfaces could help developing new prevention procedures at the initial stages of microbial adsorption, there are still too many unknown factors concerning the adhesion capacity of the main food pathogens [16]. 

The objective of this study was to find out the adhesion capacity of pathogens such as *Aeromonas hydrophila*, *Escherichia coli* O157:H7, *Salmonella *Enteritidis, and *Staphylococcus aureus* on two commonly used materials in food processing plants (polystyrene and glass). The influence of culture medium pH on the rate of adhesion by these agents at the interfaces was also simultaneously evaluated.

## 2. Material and Methods

### 2.1. Bacterial Strains, Media, and Culture Conditions

For this study, *Aeromonas hydrophila* ATCC 7966, *Escherichia coli* O157:H7 ATCC 35150, *Salmonella *Enteritidis E1347, and *Staphylococcus aureus* ATCC 29213 were selected. Cryotubes of these strains, stored at −80°C in TSB-YE (tryptic soy broth supplemented with 1% yeast extract; Difco Laboratories, Detroit, MI) containing 20% glycerol (Difco Laboratories, Detroit, MI), were thawed and the bacterial cultures were revived by two successive precultures in 10 mL of TSB-YE (1% v/v) and then incubated for 24 h at 37°C.

The harvested bacteria were washed three times and resuspended in buffers at pH 6.0, 7.0, or 8.0. A total viable count was performed for each culture and the total CFUs determined using tryptic soy agar (TSA; Difco Laboratories, Detroit, MI) were between 4 × 10^8^ and 2 × 10^9^ CFU/mL.

### 2.2. Selection of Test Surfaces and Preconditioning Procedures

Polystyrene (hydrophobic) and glass (hydrophilic) substrates were selected for the adhesion tests. Polystyrene weighing dishes (no. 25433; VWR International Inc., West Chester, PA) were used to obtain 5-cm^2^ coupons and 5-cm^2^ glass coupons were cut from microscope slides (no. 48300; VWR International Inc., West Chester, PA). Prior to physicochemical characterizations and adhesion tests, these substrates were soaked for 24 h in sodium hydroxide (1 N), washed and rinsed thoroughly eight times with deionized water (Millipore, Billerica, MA). The polystyrene coupons were sterilized for 5 min in boiling distilled water, while the glass coupons were directly autoclaved at 121°C for 15 min in bioreactors. 

### 2.3. Surface Contamination

The attachment tests were conducted in sterile bioreactors (BST Model SC60 Suspend Reactor, BioSurface Technologies Corporation, Bozeman, MT). Using a sterile clamp and under a microbiological hood, the sterile coupons were mounted on metal rods (six rods per bioreactor) in pairs, separated by a stainless steel nut. To ensure a sufficient attachment of the bacterial cells, the cultures of the pathogens in the bioreactors were left in contact with the inert surfaces for 24 h at ambient temperature (20 ± 2°C) under low agitation (90 rpm). Each experiment (bacterium-culture pH-surface type combination) was repeated three times and the means were used for the statistical analyses.

### 2.4. Rate of Adhesion of the Pathogens to Inert Surfaces

To recover the sessile cells, two coupons of each material were removed from the rods using a sterile clamp and rinsed twice in tubes containing 10 mL of saline (one tube per rinse, carefully rotating the tubes) in order to eliminate the cells that had not adhered. The substrates were then placed in a tube containing 10 mL of sterile phosphate buffer (Oxoid Ltd, Basingstoke, Hampshire, England) and all of the adhered bacterial cells were detached in a sonication bath (VWR International Inc., West Chester, PA) for 10 min. The tubes were vortexed for 30 s before the microbial counts were performed. After preparation of serial dilutions, the bacterial counts were determined by plating on TSA (tryptic soy agar; Difco Laboratories, Detroit, MI) incubated at 37°C for 24 h. The relative adhesion (%) was estimated using the following formula:

(1) $$\begin{array}{cc} & \text{Relative}\;\text{adhesion}\;\left(\text{\%}\right)\\ & \;\;=\left(\text{adhered}\;\text{bacteria}/\text{initial}\;\text{concentration}\right)\times 100. \end{array}$$

### 2.5. Scanning Electron Microscopy (SEM) Observation

 In preliminary work, the two types of noncontaminated sterile materials were gold coated and observed under a scanning electron microscope in order to characterize the microstructure of the substrates. 

After two saline rinses, the contaminated coupons were fixed by immersion in 5 mL of 2.5% glutaraldehyde (v/v) in a 0.1 M sodium cacodylate buffer (pH 7.3) and left at room temperature for 2 h. The glutaraldehyde was then removed using a Pasteur pipette and the substrates were rinsed four times by immersing them for 15 min in 0.1 M sodium cacodylate buffer (pH 7.3).

Dehydration was performed through an ascending series of ethanol (approximately 5 mL) concentrations (30%, 50%, 70%, and 80%) for 15 min for each concentration, and then three times for 15 min in 100% ethanol. The duplicates were preserved in 70% ethanol and stored at 4°C.

Dehydration was completed using CO_2_ in a critical point dryer (Model E3000 CPD, Bio-Rad, Polaron Equipment Ltd., Watford Hertfordshire, England). The samples were then mounted on an aluminum platform and covering with 8 nm of gold using a sputter coater (Cressington 108, Kurt J. Lesker Co., Clairton, PA). The substrates were observed under a scanning electron microscope (Hitachi S300N, Hitachi, Tokyo, Japan).

### 2.6. Determination of the Physicochemical Properties of the Solid and Bacteria Surfaces

For the inert material, sterile dried substrates were positioned on a microscope stage for contact angle measurements. A microsyringe (Chromatographic Specialities Inc., Brockville, Ontario, Canada) was used to place a drop (1 *μ*L) of each wetting agent—bidistilled water (Barnstead Fistreem GlassStill, England), formamide, and *α*-bromonaphthalene (Aldrich Chemical Co., Inc., Milwaukee, WI)—on the solid surfaces. The contact angles formed by these liquids were determined using a goniometer coupled with a 100 × telescope (Gaertner Scientific Corp., Chicago, Ill.). 

In each case, six measurements were taken with each liquid on each substrate to determine the surface charge of the substrates [19] as well as their hydrophilic-hydrophobic characteristics. The total surface energy (*γ* ^TOT^) of the solid substrates, the Lifshitz-van der Waals contribution to surface energies (*γ* ^LW^) and the Lewis acid-base bonds (*γ* ^AB^) were calculated using an extension of the Young-Dupré equation [20]:

(2) $$\begin{array}{cc} \left(1+\cos\;\theta \right)\times \gamma_{L}^{T} & =2[\sqrt{\left(\gamma_{S}^{\text{LS}}\times \gamma_{L}^{\text{LW}}\right)}+\sqrt{\left(\gamma_{S}^{+}\times \gamma_{L}^{-}\right)}\;\\ & \;\;\;+\sqrt{\left(\gamma_{L}^{+}\times \gamma_{S}^{-}\right)}],\\ \gamma^{\text{TOT}} & =\gamma_{s}^{\text{LW}}\times 2\sqrt{\left(\gamma_{s}^{+}\times \gamma_{s}^{-}\right)}=\gamma_{s}^{\text{LW}}\times \gamma_{s}^{\text{AB}}, \end{array}$$

where **θ** represents the angle formed by the wetting liquid on the substrate and *γ*_*L*_^*T*^, the surface tension of the wetting liquid. (*γ*_*L*_^LW^, *γ*_*L*_^−^, *γ*_*L*_^+^) and (*γ*_*S*_^LW^, *γ*_*S*_^−^, *γ*_*S*_^+^) are, respectively, the Lifshitz-van der Waals dispersion, electron donor (basic) and electron acceptor (acid) components of the wetting liquid and inert surface.

Once these parameters have been determined, for both the solid substrates (*s*) and for each bacterium (*b*) in an aqueous medium (*l*), the free energy of microbial adhesion, Δ*G*_adh_(41), was estimated according to the following formula:

(3) $$\begin{array}{cc} \Delta G_{\text{adh}} & ={\left(\sqrt{\gamma_{s}^{\text{LW}}}-\sqrt{\gamma_{b}^{\text{LW}}}\right)}^{2}-{\left(\sqrt{\gamma_{b}^{\text{LW}}}-\sqrt{\gamma_{l}^{\text{LW}}}\right)}^{2}\\ & \;-{\left(\sqrt{\gamma_{s}^{\text{LW}}}-\sqrt{\gamma_{l}^{\text{LW}}}\right)}^{2}\\ & \;+2[\sqrt{\gamma_{l}^{+}}\left(\sqrt{\gamma_{s}^{-}}+\sqrt{\gamma_{b}^{-}}-\sqrt{\gamma_{l}^{-}}\right)\;\\ & \;\;\;+\sqrt{\gamma_{b}^{-}}\left(\sqrt{\gamma_{s}^{+}}+\sqrt{\gamma_{b}^{+}}-\sqrt{\gamma_{l}^{-}}\right)\\ & \;\;\;\;-\sqrt{\left(\gamma_{s}^{+}\times \gamma_{b}^{-}\right)}-\sqrt{\left(\gamma_{s}^{-}\times \gamma_{b}^{+}\right)}]. \end{array}$$

In theory, the energy balance is favourable to bacterial adhesion if Δ*G*_adh_ < 0 and unfavourable if Δ*G*_adh_ > 0.

Bacteria physicochemical properties were determined for bacterial cultures grown at pHs 6, 7, and 8. The contact angle measurements were performed on bacterial lawns deposited on filter paper as described by Mafu et al. [19] and the total surface energy (*γ* ^TOT^) of the bacteria and the Lifshitz-van der Waals (*γ*_*S*_^LW^) contribution to surface energies as well as the Lewis acid-base bonds (*γ*_*S*_^AB^) were determined with the help of Young-Dupré relationship (2). The surface properties of the pathogens were evaluated in term of free energy of aggregation using the van der Mei et al. [21] approach  (4)

(4) $$\begin{array}{cc} \Delta G_{mwm} & =-4{\left(\sqrt{\gamma_{m}^{\text{LW}}}-\sqrt{\gamma_{w}^{\text{LW}}}\right)}^{2}\\ & \;-4(\sqrt{\left(\gamma_{m}^{+}\times \gamma_{m}^{-}\right)}+\sqrt{\left(\gamma_{w}^{+}\times \gamma_{w}^{-}\right)}\;\\ & \;\;\;\;-\sqrt{\left(\gamma_{m}^{+}\times \gamma_{w}^{-}\right)}-\sqrt{\left(\gamma_{m}^{-}\times \gamma_{w}^{+}\right)}), \end{array}$$

where (*γ*_*m*_^LW^, *γ*_*m*_^−^, *γ*_*m*_^+^) and (*γ*_*w*_^LW^, *γ*_*m*_^−^, *γ*_*m*_^+^) are, respectively, the Lifshitz-van der Waals dispersion, electron donor (base) and electron acceptor (acid) components of the microorganisms studied and those of the water. A preference for the aqueous medium, a characteristic of hydrophilic cell surfaces, is demonstrated by a Δ*G*_*mwm*_ value >0, while hydrophobic organisms, which tend to agglomerate in aqueous suspensions, are characterized by a Δ*G*_*mwm*_ < 0.

### 2.7. Statistical Analyses

The data were analysed using the GLM procedure of SAS software Version 8.0 (SAS Institute Inc., Cary, NC). An analysis of variance in the form of a factorial experiment (bacterium ∗ culture pH ∗ surface type) in complete blocks was chosen for adhesion tests. When the pH-pathogen interaction terms were significant, a one-way analysis of variance (culture pH) was performed for each of the four bacteria. The significant differences for the free energies of adhesion were detected using a one-way analysis of variance. The Duncan multiple ranges test was also used to separate means. A confidence level of *P* = .05 was chosen during analyses.

## 3. Results

### 3.1. Surfaces Characterization

**Figure 1:**
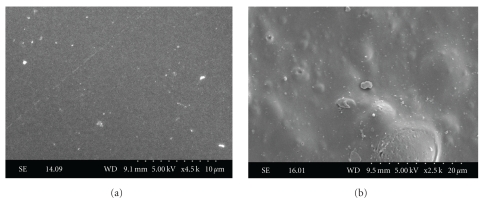
Microstructure of noncontaminated glass (a) and polystyrene (b) substrates observed under a scanning electron microscope.

Photomicrographs of uncontaminated polystyrene and glass obtained using SEM are shown in Figure 1. The substrates microstructure revealed that glass was a smooth surface (Figure 1(a)), while polystyrene had irregularities, with tiny bumps and hollows (Figure 1(b)). 

The results obtained for the surface physicochemical properties of the two substrates, determined from contact angle measurement with three solvents, are detailed in Table 1. The surfaces were characterized by a similar Lifshitz-van der Waals (*γ* ^LW^) dispersion component and by very low values for the electron acceptor parameter (*γ* ^+^). The electron donor (*γ* ^−^) and Lewis acid base capacities (*γ* ^AB^) showed considerable variability from one type of material to the other. The total surface energy (*γ* ^TOT^) measured for the polystyrene was lower than for glass. Water drops spread over the glass surface, demonstrating its hydrophilic character, consistent with the high value of the electron donor parameter. Polystyrene was characterized by extremely weak electrical properties. In addition, the angles formed by the water on the specimen were very obtuse compared with those obtained on glass, enabling us to conclude that the surface is hydrophobic.

### 3.2. Prediction of Pathogen Adhesion to Both Surfaces

The free energy of adhesion of the pathogens on bare surfaces, determined by combining the surface characteristics of the selected substrates (Table 1) and the surface properties of the bacteria (Table 4), are shown in Table 2. The Δ*G*_adh_ values of polystyrene were negative for all microorganism—culture pH—surface combinations, predicting that adhesion would be thermodynamically favourable for the polymer. This approach predicts that conditions would be unfavourable to bacterial attachment in the presence of glass, a hydrophilic surface, since the free energy of adhesion was positive in all cases. Theoretically, *A. hydrophila* and *E. coli* should exhibit weaker adsorption on both substrates at pH 6 (*P* < .05). For *S. aureus*, the rate of adhered bacteria should be highest in the case of hydrophobic surface at pH 7 (*P* < .05) and lowest in the case of hydrophilic surface at pH 8 (*P* < .05). For the two substrates studied, pH of the culture affected the theoretical affinity of *S. *Enteritidis (*P* > .05).

### 3.3. Influence of the Inert Surface and pH of the Culture on the Adhesion of Pathogenic Bacteria

At pH 6, 7, and 8, no interactions were determined between pH of culture and adhesion capability for individual bacterium (*P* > .05). The effect of each parameter therefore had to be studied independently. The type of substrate did not influence the rate of attachment of pathogens (*P* > .05), however the effect of pH of the culture varied depending on the type of microorganism considered.

*E. coli* displayed the lowest relative adhesion, regardless of environmental pH (Table 3). The affinity of *E. coli* for the solid samples was significantly influenced by pH (*P* < .05).

*A. hydrophila* on solid surfaces exhibited the same profile as *E. coli* (Table 3), however, *Aeromonas *cell had greater affinity for the two substrates than the enterohemorrhagic bacterium, especially in acid and neutral media, with the number of attached cells ranging from 45% to 53%  (*P* > .05) and from 39% to 42%  (*P* > .05), respectively. The adhesion capacity of *A. hydrophila* to inert surfaces was lower after being cultivated in an alkaline medium (*P* < .05). For *E. coli* and *A. hydrophila*, neutral pH was the best condition for attachment of these two pathogens, regardless of the type of surface.

Type of substrate and pH had no effect on the adsorption of *S. *Enteritidis to surfaces (*P* > .05). The degree of contamination of polystyrene and glass by this enterobacteria was considerably higher than that observed for the other bacteria at pH 8 (*P* < .05). At pH 6 and 7, *S*. Enteritidis had been attached to the polymer and glass equally (*P* > .05).

Also, after the contamination period, adhesion of *S. aureus* to the two types of coupons (Table 3) was lower compared to the other experimental conditions (*P* < .05). The number of organisms attached to both substrates decreased by elevating pH. At pH 8, the number of detached *S. aureus* cells was identical on both polystyrene and glass surfaces (*P* > .05).

### 3.4. Influence of the Surface Physicochemical Properties of Pathogenic Bacteria on Their Rate of Adhesion

**Figure 2:**
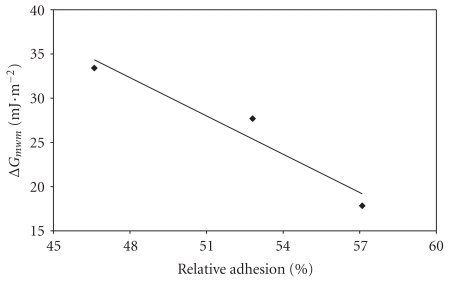
Relationship between the free energy of aggregation (*G*_*mwm*_) and the mean rate of adhesion of *Staphylococcus aureus *to the surfaces studied (*r* ^2^ = 0.94).

Figure 2 demonstrates the mean relative adhesion rate of each bacterium as a function of the component of their surfaces. In fact, since the solid surfaces had no effect on the concentration of adhered cells (*P* > .05), the mean between the relative adhesion to polystyrene and to glass could be calculated in order to obtain an overall adhesion rate. Only an high correlation coefficient (*r* ^2^ = 0.94) between the free energy of aggregation of *S. aureus* and its mean rate of adhesion to the substrates has been found (Figure 2). The greater the hydrophilic character displayed by this bacterium, the lower its tendency to attach to inert surfaces.

### 3.5. Scanning Electron Microscopy Observations

**Figure 3:**
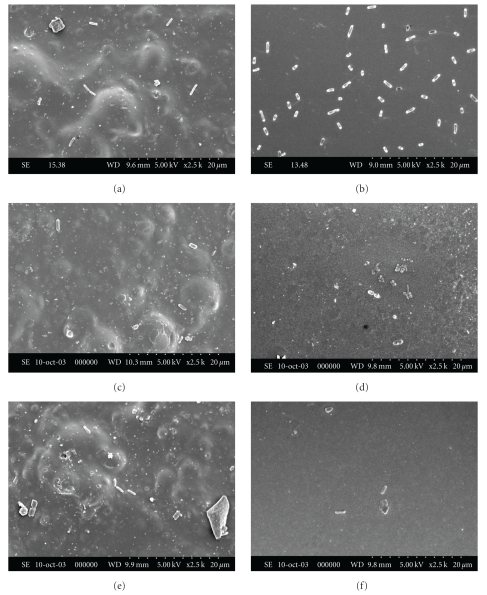
Attachment of *Salmonella enteritidis* to polystyrene ((a), (c), and (e)) and to glass ((b), (d), and (f)) at pH 6 ((a) and (b)), pH 7 ((c) and (d)) and pH 8 ((e) and (f)).

**Figure 4:**
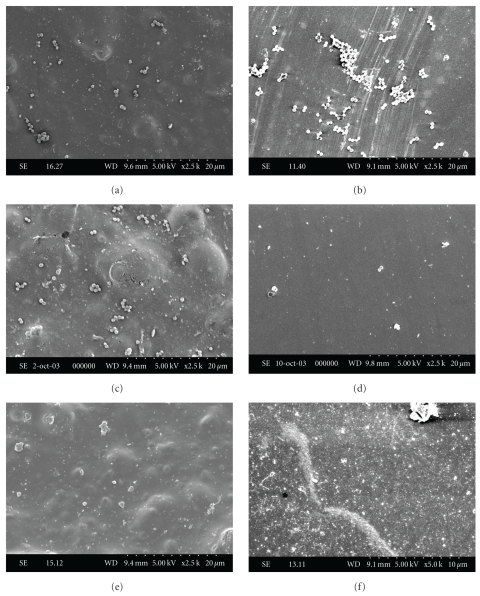
Attachment (at different pH levels) of *Staphylococcus aureus* to polystyrene ((a), (c), and (e)) and to glass ((b), (d), and (f)) at pH 6 ((a) and (b)), pH 7 ((c) and (d)), and pH 8 ((e) and (f)).

On both types of surfaces, porous and nonporous, a very low number of *E. coli* and dispersed cells of *A. hydrophila *were observed (data not shown). That is why no photomicrographs of these microorganisms have been provided. *S. *Enteritidis cells were generally isolated or formed small clusters, regardless of the adhesion conditions (Figure 3). Their dispersion was more uniform at pH 6 than at the other pH values (Figures 3(a) and 3(b)). *S. aureus* invaded the abiotic surfaces in the form of single cells, small chains, or cell aggregates (Figure 4). It was more difficult to observe staphylococci on polystyrene and glass at pH 8 (Figures 4(e) and 4(f)). As demonstrated by the present results, it becomes clear that in all case, no visible extracellular material was observed on both food contact surfaces.

## 4. Discussion

Bacterial adhesion to surfaces and biofilm formation are complex phenomena influenced by a number of factors. In this study, three of these factors, namely, microbial strains, culture pH, and type of surface, were analysed. Polystyrene and glass were chosen as surfaces, respectively, hydrophobic and hydrophilic, for the attachment and biofilm formation tests. 

Scanning electron microscopy observation (Figures 3 and 4) showed that all the pathogens had the capacity to adhere to both types of surface. Although the cavities and distortions of the samples observed under the scanning electron microscope increase the specific contact area for bacteria, the differences in surface irregularities between both solid surfaces had no observable impact on the relative adhesion of the microorganisms. These results are consistent with the findings of Mafu et al. [22], who found no correlation between the microstructure of the materials and the capacity of bacteria to adhere to surfaces. Also in this study, the attachment of solid surfaces by microorganisms was influenced solely by culture pH, which concurs with the work of Husmark and Ronner [23] as well as that of Herald and Zottola [24]. The absence of fimbriae and curli [25, 26] might explain the low contamination of *E. coli* observed in this study as well as in other experiments conducted on the contamination of polymers by serotype O157 [24, 27, 28]. 

The data from these experiments are not in agreement with the predictions deduced from the free energies of adhesion (Δ*G*_adh_) of pathogens on polystyrene (Table 2). These energy characteristics demonstrated that conditions for adsorption of all microorganisms were thermodynamically favourable on polystyrene (Δ*G*_adh_< 0) and unfavourable on glass (Δ*G*_adh_ > 0) surfaces. The bacterial cultures remained in contact with the inert surfaces for 24 hours. After this contact time, the adhesion stage tends to be irreversible. If the exposure time had been shorter (maximum of four hours, which corresponds to irreversible bacterial adsorption), the predictions for the adhesion, based on Δ*G*_adh_, might have proven valid. In fact, the equation used to predict bacterial adhesion to surfaces (Δ*G*_adh_) does not take into consideration the exopolysaccharides that might be produced for the irreversible attachment of microorganisms.

In another attempt to explain adhesion detection with Δ*G*_adh_ values >0, we endeavoured to determine whether the surface properties of the infectious agents influenced their adhesion rate. Thus, the free energy of aggregation of *S. aureus* could be correlated to the degree of attachment of the cocci (Figure 2). Gilbert et al. [29] also demonstrated that the adhesion of *Staphylococcus epidermidis* to glass, unlike *Escherichia coli*, was negatively correlated to the microorganism's hydrophilicity and surface electronegativity. However, the physicochemical characteristics of the inert surfaces and of the other three pathogens examined throughout this study did not shed any light on their adhesion capacity. The explanation may lie in the composition of the suspension medium of the microorganisms, which was not taken into account in the estimation of PS and glass free energy.

Indeed, conditioning the substrates with compounds from the suspension medium can increase or decrease subsequent bacterial adhesion [23, 30, 31]. These ionic substances located at the microorganism-surface interface can change ionic strength which is a critical factor in adhesion. Some of these elements can reduce the repulsive force between the bacterium and the solid surfaces [32, 33], due to electrostatic interactions, by masking charges of the same sign. Thus, several stress responses are associated with the appearance of macromolecular agents in the medium. Many microorganisms exposed to acidic and alkaline stress synthesize polysaccharides, peptides, or heat-stable proteins [10, 34], which play an active role in induction of tolerance to pH stress. It should also be noted that some bacteria produce biosurfactants that inhibit their attachment. For instance, synthesis by *S. aureus* of a surfactant, the toxin *δ*, inhibits the action of D-alanine [12], limiting cell adhesion to polystyrene [35].

Previous studies have also demonstrated the important roles played by cell organelles and bacterial mobility in transport and adhesion to various types of surfaces. *Pseudomonas fluorescens* strains without flagella exhibited a weak capacity to attach to surfaces and develop a biofilm [7]. Flagella apparently play an essential role during the initial reversible stages of attachment by overcoming the repulsive forces. However, the nonmotile bacterium *S. aureus* adhered to the tested substrates at pH 6 in greater numbers than *E. coli* O157:H7 and *A. hydrophila* (Table 3), which are motile microorganisms. The presence of flagella is apparently more important when the velocity of the environmental liquid is high [4, 36]. McClaine and Ford [37] demonstrated that rotation of the flagellum caused detachment of fixed motile bacteria when flow rates were low (0.02 mL·min^−1^), while their adhesion was strengthened at high flow rates (2 mL·min ^−1^). 

Although the irreversible attachment of microorganisms is often associated with the production of exopolysaccharides, no extracellular matrix was detected by scanning electron microscopy, regardless of experimental conditions. This indicates that the presence of polymers is not necessary for permanent adhesion of microorganisms to a surface. The visible filaments attached to the substrates may be dependent on the suspension medium. Maximum polysaccharide production is generally associated with an environment deficient in essential nutrients, such as carbon, nitrogen, calcium, and iron [26, 38]. Contact time and environmental temperature are other factors that influence the formation of a three-dimensional network [39, 40]. However, these appendices are crucial for biofilm consolidation and their resistance to environmental stresses [41]. 

Several molecules from the culture media can contribute to produce a conditioning film at the surface of the substrates [42]. These molecules include proteins, which are block copolymers presenting hydrophobic as well as hydrophilic sections. It is thus possible for such molecules to adhere to hydrophilic substrates such as glass, resulting in an hydrophobization of the surface. This phenomena would lead bacteria presenting the same adhesion trends on both PS and hydrophobically coated glass.

## 5. Conclusion

The results of this work indicate that pathogens could adapt to various pH levels of cultural media and adhere indifferently to inert polystyrene and glass surfaces, regardless of their hydrophilic or hydrophobic nature which leads to biofilm formation and increases the possibility of resistance to sanitizing agents. Therefore, to minimize surfaces contaminations, one should be aware of the influence of different environmental conditions which affects survival of bacteria in order to reduce their incidence in food systems. The operations must be carried out promptly and regularly (between two production cycles, for example), since they can effectively detach and eliminate the adhered bacteria when they have not yet formed a biofilm, thereby preventing contamination of raw materials and finished products and protecting consumer health.

## Figures and Tables

**Table 1:** Contact angles (°) and surface energies (mJ · m^−2^) of solid surfaces.

Surface	*θ*_*w*_(°)^1^	*θ*_*F*_(°)^1^	*θ*_*α*−*B*_(°)^1^	*γ*_*S*_^LW^	*γ*_*S*_^+^	*γ*_*S*_^−^	*γ*_*S*_^AB^	*γ* ^TOT^
Polystyrene	95.5 ± 1.2	59.3 ± 2.4	9.8 ± 1.3	43.8	0.2	0.0	0.0	43.8
Glass	13.6 ± 1.7	12.9 ± 2.1	17.0 ± 0.9	42.5	0.8	53.4	12.8	55.3

^1^ *θ*_*w*_, *θ*_*F*_, and *θ*_*α*−*B*_ are on average the angles formed by water, formamide, and *α*-bromonaphthalene, respectively,

where *γ*_*S*_^LW^, *γ*_*S*_^+^, *γ*_*S*_^−^, *γ*_*S*_^B^, and *γ* ^TOT^ are, respectively, the Lifshitz-van der Waals dispersion component, electron acceptor (acid) and electron donor (basic) parameters, Lewis acid-base bonds, and the total surface energy of the solid substrates.

**Table 2:** Free energy of adhesion (mJ · m^−2^) of the four pathogens to polystyrene (PS) and to glass (GS) as a function of culture pH.

Bacterium	pH	Δ*G*_adh_ PS^1^	Δ*G*_adh_ GS^1^
*Aeromonas hydrophila*	6	−35.9 ± 1.4^b^	18.0 ± 0.2^b^
	7	−37.7 ± 1.0^b^	18.2 ± 0.3^b^
	8	−38.0 ± 0.1^b^	18.0 ± 0.1^b^

*Escherichia coli* O157:H7	6	−38.8 ± 1.3^a^	17.3 ± 0.3^b^
	7	−39.7 ± 0.4^a^	17.3 ± 0.0^b^
	8	−39.0 ± 1.1^a^	17.3 ± 0.1^b^

*Salmonella *Enteritidis	6	−40.9 ± 0.5^a^	17.1 ± 0.4^a^
	7	−42.8 ± 0.8^a^	16.9 ± 0.0^a^
	8	−42.8 ± 1.4^a^	16.9 ± 0.0^a^

*Staphylococcus aureus*	6	−39.6 ± 0.4^a^	16.1 ± 0.8^c^
	7	−43.2 ± 1.1^b^	17.0 ± 0.1^bc^
	8	−41.4 ± 0.4^ab^	18.3 ± 0.0^a^

^1^Δ*G*_adh_ PS and Δ*G*_adh_ GS are the free energies of adhesion of the bacteria to polystyrene and glass, respectively.

^a–c^ In a column, for each given bacterium, values with the same letter are not significantly different (*P* > .05).

**Table 3:** Effect of culture pH on the adhesion of pathogenic bacteria to polystyrene and glass.

Organism	pH	Relative adhesion (%)	
		Solid surfaces	
		Polystyrene	Glass
*A. hydrophila*	6	48.8 ± 0.4	50.8 ± 2.6
	7	50.6 ± 1.9	54.2 ± 3.5
	8	45.0 ± 5.1	44.6 ± 4.3

*E. coli *O157:H7	6	39.4 ± 2.0	40.7 ± 0.6
	7	42.1 ± 2.4	42.1 ± 0.5
	8	39.3 ± 2.1	39.5 ± 1.1

*S. enteritidis*	6	54.6 ± 5.3	55.3 ± 2.5
	7	56.7 ± 3.4	55.1 ± 3.0
	8	62.11 ± 2.3	54.31 ± 3.8

*S. aureus*	6	58.6 ± 3.2	55.6 ± 3.5
	7	54.4 ± 5.6	51.1 ± 8.3
	8	46.61 ± 4.2	46.71 ± 1.8

**Table 4:** Surface tension and hydrophobicity (mJ · m^−2^) of the four pathogens determined from contact angle measurement.

Organism	pH	*γ*_*S*_^LW^	*γ*_*S*_^+^	*γ*_*S*_^−^	*γ*_*S*_^AB^	*γ* ^TOT^	Δ*G*_mwm_^1^
*A. hydrophila*	6	35.3 ± 1.0^a^	1.9 ± 0.2^b^	53.0 ± 0.4^a^	20.0 ± 1.0^b^	55.3 ± 0.1^a^	26.3 ± 0.2^b^
	7	35.4 ± 0.7^a^	1.5 ± 0.1^b^	54.1 ± 0.3^a^	18.2 ± 0.8^b^	53.6 ± 0.1^a^	28.7 ± 0.4^a^
	8	35.8 ± 0.4^a^	1.7 ± 0.0^b^	53.6 ± 0.0^a^	18.1 ± 0.0^b^	53.9 ± 0.4^a^	27.8 ± 0.3^ab^

*E. coli 0157:H7*	6	37.4 ± 0.1^bc^	1.5 ± 0.2^a^	52.3 ± 1.3^a^	17.9 ± 1.3^a^	55.3 ± 1.2^a^	24.9 ± 0.8^c^
	7	38.5 ± 0.1^a^	1.4 ± 0.1^a^	54.0 ± 0.1^a^	17.3 ± 0.3^a^	55.8 ± 0.3^a^	26.2 ± 0.0^b^
	8	38.2 ± 0.6^ab^	1.6 ± 0.2^a^	53.5 ± 0.4^a^	17.9 ± 1.0^a^	56.0 ± 0.3^a^	25,6 ± 0.4^bc^

*S. enteritidis*	6	36.1 ± 0.7^d^	1.7 ± 0.0^a^	49.8 ± 0.8^b^	18.3 ± 0.3^a^	54.4 ± 0.5^a^	23.0 ± 1.4^a^
	7	38.1 ± 0.0^ab^	1.0 ± 0.1^a^	51.1 ± 0.2^ab^	14.0 ± 0.7^a^	52.1 ± 0.7^a^	25.1 ± 0.7^a^
	8	38.6 ± 0.3^a^	1.0 ± 0.2^a^	52.0 ± 0.3^a^	14.2 ± 1.3^a^	52.8 ± 1.0^a^	25.6 ± 0.9^a^

*S. aureus*	6	37.5 ± 0.3^b^	1.6 ± 0.1^a^	46.0 ± 3.5^b^	17.0 ± 0.3^a^	54.5 ± 0.0^a^	17.8 ± 4.4^c^
	7	39.7 ± 0.1^a^	0.9 ± 0.2^b^	54.6 ± 1.3^a^	14.2 ± 1.1^b^	53.8 ± 1.0^a^	27.7 ± 2.1^ab^
	8	36.5 ± 0.1^b^	1.0 ± 0.1^b^	54.7 ± 0.5^a^	15.0 ± 0.4^b^	51.6 ± 0.3^b^	33.4 ± 0.8^a^

^1^  Free energy of aggregation of the microorganisms in water where *γ*_*S*_^LW^, *γ*_*S*_^+^, *γ*_*S*_^−^, *γ*_*S*_^*B*^, and *γ* ^TOT^ are, respectively, the Lifshitz-van der Waals contribution energies, electron acceptor (acid), and electron donor (basic) components of the wetting agent and bacterial lawn, Lewis acid-base bonds and the total surface energy of the bacteria.

^a–d^ In a column, for a given bacterium, values with the same letter are not significantly different (*P* > .05).
